# Psychometric properties of the PROMIS Preference score (PROPr) in patients with rheumatological and psychosomatic conditions

**DOI:** 10.1186/s41927-022-00245-3

**Published:** 2022-03-07

**Authors:** C. P. Klapproth, F. Fischer, M. Merbach, M. Rose, A. Obbarius

**Affiliations:** 1 Health Outcomes Research, Department of Psychosomatic Medicine, Center for Internal Medicine and Dermatology, Charité - Universitätsmedizin Berlin, Charitéplatz 1, 10117 Berlin, Germany; 2 Department of Quantitative Health Sciences, University of Massachusetts Medical School, Worcester, USA; 3 Dornsife Center for Self-Report Science, University of Southern California, Los Angeles, USA

**Keywords:** Patient reported outcomes, Quality of life, Pain, Health status, Outcome measures, QALY, PROPr, EQ-5D, ICER

## Abstract

**Background:**

The PROMIS Preference score (PROPr) is a new generic preference-based health-related quality of life (HRQoL) score that can be used as a health state utility (HSU) score for quality-adjusted life years (QALYs) in cost-utility analyses (CUAs). It is the first HSU score based on item response theory (IRT) and has demonstrated favorable psychometric properties in first analyses. The PROPr combines the seven PROMIS domains: cognition, depression, fatigue, pain, physical function, sleep disturbance, and ability to participate in social roles and activities. It was developed based on preferences of the US general population. The aim of this study was to validate the PROPr in a German inpatient sample and to compare it to the EQ-5D.

**Methods:**

We collected PROPr and EQ-5D-5L data from 141 patients undergoing inpatient treatment in the rheumatology and psychosomatic departments. We evaluated the criterion and convergent validity, and ceiling and floor effects of the PROPr and compared those characteristics to those of the EQ-5D.

**Results:**

The mean PROPr (0.26, 95% CI: 0.23; 0.29) and the mean EQ-5D (0.44, 95% CI: 0.38; 0.51) scores differed significantly (d = 0.18, p < 0.001). Compared to the EQ-5D, the PROPr scores were less scattered across the measurement range which has resulted in smaller confidence intervals of the mean scores. The Pearson correlation coefficient between the two scores was r = 0.72 (p < 0.001). Both scores showed fair agreement with an Intraclass Correlation Coefficient (ICC) of 0.48 (p < 0.05). The PROPr and EQ-5D demonstrated similar discrimination power across sex, age, and conditions. While the PROPr showed a floor effect, the EQ-5D showed a ceiling effect.

**Conclusion:**

The PROPr measures HSU considerably lower than the EQ-5D as a result of different construction, anchors and measurement ranges. Because QALYs derived with the EQ-5D are widely considered state-of-the-art, application of the PROPr for QALY measurements would be problematic.

**Supplementary Information:**

The online version contains supplementary material available at 10.1186/s41927-022-00245-3.

## Background

The global burden of autoimmune diseases, including many rheumatological conditions, has grown substantially over the past decades. Its costs for both national health plans and society have increased accordingly [1–3]. Therefore, in light of budget constraints to the national health plans, which are additionally stressed by the global COVID-19 pandemic, researchers in rheumatology will need to prove the value of new and more sophisticated treatments to Health Technology Assessement (HTA) agencies, who are responsible for reimbursement decision making and thus patients’ access to new treatments.

These decisions rely on the new treatment’s value, or cost-effectiveness, measured by the incremental cost effectiveness ratio (ICER) in costs per quality-adjusted life years (QALYs) gained compared to the standard of care (SOC) [4–6]:

$${\text{ICER}} = \frac{{{\text{Costs}} \,\left( {{\text{new}}\,{\text{treatment}}} \right) - {\text{Costs}} \,\left( {{\text{SOC}}} \right)}}{{{\text{QALY}} \,\left( {{\text{new}}\,{\text{treatment}}} \right) - {\text{QALY}}\, \left( {{\text{SOC}}} \right)}} = \frac{{\Delta {\text{Costs}}}}{{\Delta {\text{QALY}}}}$$

A QALY is the number of remaining life years multiplied by a health state utility (HSU or u) value between 0 and 1, where 0 represents death and 1 represents perfect health [7]. Many HTA agencies adopted an ICER threshold. For example, the National Institute for Health and Care Excellence (NICE) in England and Wales has a threshold of 30,000 pounds per QALY gained; new treatments are not reimbursed if the costs exceed this threshold [8–10]. It is therefore crucial for the patients’ access to new treatments that HSU assessments are valid, precise, reliable, and responsive to change.

The HSU is obtained from preference-based health-related quality of life (HRQoL) scores, such as the Short Form-6 Dimensions (SF-6D) or the EuroQol EQ-5D index value (EQ-5D) [11]. The selection of the specific score (e.g., SF-6D or EQ-5D) has an impact on the ICER, as they differ in terms of health dimensions, number of measurement levels, valuation technique, and reference population [5]. The EQ-5D, for example, includes five dimensions: mobility, self-care, usual activities, pain/discomfort, and depression/anxiety [12]. Each is measured with one item on five levels, describing 5^5^ or 3125 health states. These descriptive health states are assigned a value between 0 and 1 or even below 0 (“worse than dead”) by preference elicitation methods such as standard gamble (SG) or time trade-off (TTO). Note that the value that is assigned to health states reflects the preferences of the general population and therefore overwhelmingly healthy people, not the preferences of patients who merely describe their health states through these measures [11, 13].

The EQ-5D was endorsed by many HTA agencies [4, 8]. It demonstrates good psychometric performance in terms of construct validity and responsiveness in some conditions (e.g., rheumatoid arthritis or depression). However, it is criticized for performing poorly in other conditions (e.g., mental health conditions) [11]. Results were inconsistent in some conditions (i.e., COPD and cardiovascular diseases) [11].

Additionally, existing HSU scores were criticized for their imprecise measurement in individuals and small samples, a limited range of measurement indicated by ceiling effects, and double-barreled items (e.g., EQ-5D anxiety/depression) [14].

These shortcomings motivated the development of a new preference-based HRQoL score based on instruments from the Patient Reported Outcome Information System (PROMIS), called the PROMIS Preference (PROPr) score [14–19]. PROMIS is a broad collection of patient-reported outcomes or constructs such as physical function or depression using large item banks, based on item response theory (IRT). IRT allows to measure and compare the respective constructs on a common scale (T-score metric), even when using different sets of items. This allows to tailor the assessment to the expected scores of the population under investigation. So, each domain can be measured by one, two, four or more items to avoid floor or ceiling effects or to increase measurement precision [20–24]. Seven of these PROMIS domains where selected for the PROPr: cognition, depression, fatigue, pain interference, physical function, sleep disturbance, and ability to participate in social roles and activities. The PROPr was valuated with the preferences of the US population using online SG [14–19]. It leverages the excellent psychometric properties of the PROMIS item banks: high validity and reliability, avoidance of floor and ceiling effects, high sensitivity to change, high precision, and small sample size requirements [20–24]. Therefore, the PROPr has the potential to overcome limitations such as imprecision in small samples and ceiling effects. The validity of the PROPr has already been shown in US general population samples but not yet in patient samples or samples from countries outside the US [16, 18].

In this study, we seek (1) to assess the validity of the PROPr in a sample of patients with rheumatological and psychosomatic conditions, (2) to compare the measurement properties of the PROPr and the EQ-5D, which is known to perform well in this patient group [11], (3) to investigate the causes of the differences between the two scores by analyzing their ceiling and floor effects and (4) to discuss the consequences for the ICER that arise from the different conceptualizations of the two scores.

## Patients and methods

### Samples

We conducted a cross-sectional study and invited patients undergoing inpatient treatment at the Department of Rheumatology and Immunology and the Department of Psychosomatic Medicine at Charité—Universitätsmedizin Berlin to participate. We believe that this patient group may be a good indicator for the current state-of-the-art treatment, or standard of care (SOC), of severer cases. Patients participated between March 2018 and August 2018 (rheumatology department) and between October 2018 and June 2019 (psychosomatic department). Informed consent was obtained from all patients directly as none of them had legal guardians. A set of various HRQoL questionnaires was administered to the patients, including 14 PROPr items, the 5 EQ-5D-5L items, and sociodemographic questions. In rheumatology, items were administered in a paper-and-pencil form. Data were collected electronically in the psychosomatic department. There were no inclusion or exclusion criteria regarding condition, age, sex or kind of treatment applied. Reasons for exclusion were previous participation in this study, impaired vision, illiteracy, language barrier, and inability to use the tablet.

This study was approved by Charité’s Ethics Committee (EA/133/17) and was conducted in accordance with the Declaration of Helsinki.

### Measures

#### PROMIS Preference score (PROPr)

The PROPr is a new preference-based HRQoL score developed and copyrighted by the PROMIS Health Organization. It is based on the PROMIS framework and is therefore the first HSU based on IRT. It aggregates seven PROMIS domains: cognition, depression, fatigue, pain, physical function, sleep disturbance, and ability to participate in social roles and activities. We used the two recommended items from each of the 7 domains (i.e., 14 items in total, Table 1) [14–19]. Both items for cognition and ability to participate in social roles and activities and one fatigue item had not yet passed the translation process into German at the time of our survey; therefore, we used the preliminary translations. Since then, the final versions of three of these five items were released and were very similar to the preliminary versions (see Additional file 1: Table S1). Note that potentially, due to the IRT property, any other different selection of items from the PROMIS item banks could be used to measure the PROPr domains.

**Table 1:** PROPr domains and items

PROPr domain	Item code	Item
Cognition	PC6r	I have been able to concentrate
	PC27r	I have been able to remember to do things, like take medicine or buy something I needed
Depression	EDDEP36	I felt unhappy
	EDDEP45	I felt that nothing was interesting
Fatigue	FATIMP21	How often were you too tired to take a bath or shower?
	FATIMP20	How often did you feel tired?
Pain	PAININ29	How often was your pain so severe you could think of nothing else?
	PAININ24	How often was pain distressing to you?
Physical function	PFA16r1	Are you able to dress yourself, including tying shoelaces and buttoning up your clothes?
	PFC13r1	Are you able to run 100 yards (100 m)?
Sleep disturbance	Sleep110	I got enough sleep
	Sleep50	I woke up too early and could not fall back to sleep
Ability to participate in social roles and activities	SRPPES31_CaPS	I have trouble taking care of my regular personal responsibilities
	SRPPER04_CaPS	I have trouble participating in recreational activities with others

Each of these items is measured on five levels (e.g., “never”, “rarely”, “sometimes”, “often”, “always” or “not at all”, “a little bit”, “somewhat”, “quite a bit”, and “very much”) and, except for physical function, refers to the past 7 days. The responses (1 to 5 on a Likert scale) were transformed into PROMIS theta scores (mean = 0 ± SD = 1) or T-Scores (mean = 50 ± SD = 10) (http://www.healthmeasures.net/score-and-interpret/calculate-scores). For cognition, physical function, and ability to participate in social roles and activities, higher T-Scores (thetas) indicate more desirable outcomes. For depression, fatigue, pain, and sleep disturbance, lower T-Scores (thetas) indicate more desirable outcomes.

Theta scores were fed into a multi-attribute utility (MAUT) function to obtain the PROPr, ranging from − 0.022 to 1.00. Negative values indicate a health state that is perceived as “worse than dead”. The MAUT function was derived from the preferences of a representative sample of the US population by online SG in 2016 and is available online as the R code used in this study [18, 25].

#### EQ-5D-5L index value

The EQ-5D is a preference-based instrument to measure HRQoL that consists of five health dimensions: mobility, self-care, usual activities, pain/discomfort, and anxiety/depression. The EQ-5D-5L differentiates five levels for each dimension: “No problems” (score: 1), “Slight problems” (2), “Moderate problems” (3), “Severe problems” (4), and “Extreme problems” (5). The frame of reference is “Today”. All items and response options yield 5^5^ or 3125 different health states. The value assigned to each health state was determined through cTTO following the standardized EuroQol VT protocol [26–28]. We use the US value set of 2017, as the PROPr was also valuated in US preferences only one year earlier (2016, see above), avoiding systematic differences due to different valuation populations. A health state of 11,111 (i.e., each of the five items is answered with ‘1’) has an HSU of 1.00 (perfect health). The worst health state, 55,555, corresponds to an HSU of -0.573. Negative HSUs are considered “worse than dead”.

### Statistical analysis

First, we investigated the criterion validity between the PROPr and the EQ-5D. Pearson correlations were used to measures associations: r > 0.7 refers to high, r > 0.5 refers to intermediate and r < 0.5 refers to low associations [29]. Furthermore, we calculated the Intraclass Correlation Coefficient (ICC) as a measure of agreement: > 0.75 is defined as excellent, > 0.6 as good, > 0.4 as fair, and < 0.4 as poor agreement [30].

Second, for convergent validity, we investigated how differences between the two scores are affected by age, sex, and condition. We modeled HSU using a linear regression model with interaction terms to statistically test whether the mean differences depend on the instrument alone or whether they are influenced by age, sex or condition. The regression equations were defined as HSU = intercept + instrument + age/sex/condition + instrument*age/sex/condition. We hypothesized that there is a significant instrument effect, as indicated by earlier research.

Third, we compared ceiling and floor effects on subscale and score levels. If more than 15% of the sample scored within the top (bottom) 20% of the respective measurement scale, this defined a significant ceiling (floor) effect. This is the equivalent of scoring the highest or lowest score on a 5-point Likert scale that was used at the item level. Moderate ceiling (floor) effects were defined by > 10%, minor by > 5%, and negligible by < 5% of the sample [31].

We used Microsoft Excel 2016, IBM SPSS Statistics version 25 and R 4.0.0 with packages eq5d version 0.7.0, ggplot2 version 3.3.0, psych version 1.9.12.31, tidyverse 1.3.0, lme4 1.1–23, lmerTest 3.1–3, reshape2 version 1.4.4, and PearsonDS 1.2 for analyses.

## Results

### Sample

We used a combined patient sample of 141 patients receiving inpatient diagnostics and treatment in our rheumatology and psychosomatic departments. In the rheumatology (psychosomatic) department, of 236 (157) patients screened, 10 (14) patients were not eligible to participate. A total of 162 (90) patients agreed to participate and received questionnaires after informed consent was obtained. A total of 118 (58) sets were returned, while 44 (32) participants either declined to participate after receiving questionnaires or were discharged early and did not return a questionnaire. In rheumatology, of these 118 returned sets, 83 completed all PROPr and EQ-5D items needed for this study. Skipped items may be explained by a high response burden, as our items were part of a larger survey comprising various HRQoL questionnaires. Tablet administration in the psychosomatic department prevented participants from skipping items, as only complete questionnaires were submitted to the database.

Sample characteristics can be obtained from Table 2. In both samples, two-thirds of participants were female, and almost two-thirds lived with a partner. More than 85% of both samples were of German nationality. Ethnicity was not assessed, as it is neither in German population census for historical reasons [32]. Approximately one-third had a masters, bachelors or doctoral degree. On average, Rheumatology patients had a higher level of education than psychosomatic patients. One out of five was working part- or full-time, while more than one-third was unable to work for health reasons. Patients from the rheumatology department were more likely to be unable to work for health reasons than patients from the psychosomatic department. In the rheumatology department, arthritis (rheumatoid arthritis, psoriasis arthritis, ankylosing spondylitis, and gout arthritis), systemic sclerosis (SScl), systemic lupus erythematosus (SLE), and vasculitis (small and large vessel vasculitis, polymyalgia rheumatica) were the most frequent conditions. In the psychosomatic department, all patients had persistent somatoform pain disorder (PSPD, ICD-10 code F45.4) as the primary condition.

**Table 2:** Sample characteristics

Variable	Total sample n = 141	Rheumatology n = 83	Psychosomatic n = 58
Age in years, mean ± SD (range)	53.3 ± 16.2 (19–86)	55.2 ± 17.4 (19–86)	50.6 ± 14.1 (24–80)
*Sex, n (%)*			
Female	95 (67.4)	58 (69.9)	37 (63.8)
Male	46 (32.6)	25 (30.1)	21 (36.2)
*Nationality*			
German	125 (88.7)	72 (86.7)	53(91.7)
Non-German	7 (4.9)	7 (8.4)	0 (00.0)
Unknown	9 (6.4)	4 (4.8)	5 (8.6)
*Living status, n (%)*			
With partner	88 (62.4)	53 (63.9)	35 (60.3)
Single	39 (27.7)	24 (28.9)	15 (25.9)
Other	8 (5.7)	5 (6.0)	3 (5.2)
Unknown	6 (4.2)	1 (1.2)	5 (8.6)
*Educational level (ISCED 1997*^*1*^*), n (%)*			
Doctoral degree or equivalent	3 (2.1)	2 (2.4)	1 (1.7)
Bachelor’s/Master’s degree or equivalent	45 (32.0)	31 (37.3)	14 (24.1)
Degree of post-secondary/tertiary education	43 (30.5)	25 (30.1)	18 (31.0)
Degree of secondary education	12 (8.5)	8 (9.6)	4 (6.9)
Degree of primary education	28 (19.9)	15 (18.1)	13 (22.4)
Without	4 (2.8)	1 (1.2)	3 (5.2)
Unknown	6 (4.2)	1 (1.2)	5 (8.6)
*Work status, n (%)*			
Full-time	20 (14.2)	11 (13.3)	9 (15.5)
Part-time	8 (5.7)	7 (8.4)	1 (1.7)
Seeking employment	4 (2.8)	1 (1.2)	3 (5.2)
Not employed (student, retired, freelancer)	48 (34.0)	20 (24.1)	28 (48.3)
Unable to work for health reasons	51 (36.2)	36 (43.4)	15 (25.9)
Unknown	10 (7.1)	5 (6.0)	5 (8.6)
*Primary conditions, n (%)*			
Persistent somatoform pain disorder (PSPD)^2^			58 (100.0)
Arthritis		19 (22.9)	
Systemic sclerosis (SScl)		17 (20.5)	
Systemic lupus erythematosus (SLE)		13 (15.7)	
Vasculitis		11 (13.3)	
Others^3^		11 (13.3)	
Sjogren’s syndrome (SS)		9 (10.8)	
Ormond’s disease		3 (3.6)	
*Domain scores, M (SD)*			
EQ-5D-5L Mobility^4^	2.50 (1.14)	2.34 (1.15)	2.72 (1.09)
EQ-5D-5L Self-care^4^	1.67 (0.92)	1.63 (0.92)	1.72 (0.93)
EQ-5D-5L Usual Activities^4^	2.85 (1.26)	2.64 (1.30)	3.16 (1.15)
EQ-5D-5L Pain/Discomfort^4^	3.02 (1.10)	2.60 (1.01)	3.62 (0.95)
EQ-5D-5L Anxiety/Depression^4^	2.28 (1.15)	1.87 (0.93)	2.88 (1.19)
PROPr Cognition^5^	48.18 (6.65)	49.68 (7.13)	46.04 (5.52)
PROPr Depression^6^	55.39 (9.90)	53.17 (9.19)	58.57 (10.08)
PROPr Fatigue^6^	57.26 (9.25)	55.13 (9.70)	60.31 (7.67)
PROPr Pain^6^	62.03 (8.16)	59.72 (8.90)	65.33 (5.56)
PROPr Physical Function^5^	41.54 (8.59)	42.12 (8.58)	40.69 (8.60)
PROPr Sleep Disturbance^6^	52.32 (8.24)	50.59 (8.42)	54.79 (7.35)
PROPr Ability to Participate in Social Roles^5^	44.48 (8.62)	46.57 (9.00)	41.24 (6.91)
*Health States Utilities, M (95%CI) [Range]*			
PROPr	0.26 (0.23; 0.29) [− 0.004; 0.955]	0.32 (0.27; 0.36) [0.009; 0.995]	0.18 (0.15; 0.21) [− 0.004; 0.53]
EQ-5D-5L index value (US 5L Value Set)	0.44 (0.38; 0.51) [− 0.425; 1.00]	0.57 (0.50;0.65) [− 0.425; 1.00]	0.26 (0.17; 0.36) [− 0.329; 1.00]

^1^International Standard Classification of Education

^2^ ICD-10 code F45.4 (International Classification of Disease, 10th revision)

^3^Others include: keratitis, orbital tip syndrome, Still’s disease, other myopathy, fever of unknown origin, neoplasm of unknown behavior in the craniopharyngeal duct, acute otitis media, vesicular pityriasis

^4^Standard measure to assess general health-related quality of Life (HrQoL) developed by the EuroQoL group, 1 = no impairment, 5 = full impairment

^5^PROPr PROMIS domains in theta T-score; 50 = population average; −/+ 10 SD worse/better than population average, higher values indicate better function

^6^PROPr PROMIS domains in theta T-score; 50 = population average; −/+ 10 SD worse/better than population average, lower values indicate better function; n = number; M = Mean; SD = standard deviation; CI = confidence interval

### Criterion validity and distribution

Table 2 shows that patients from the psychosomatic department reported a lower HRQoL than patients from the rheumatology department in all EQ-5D and PROPr domain scores and had significantly lower HSU scores. The PROPr showed narrower confidence intervals than the EQ-5D in both samples. For the remaining analysis, we used the total sample. The mean PROPr (0.26, 95% CI: 0.23; 0.29) score was significantly lower (d = 0.18, p < 0.001) than the mean EQ-5D (0.44, 95% CI: 0.38; 0.51). The correlation was r = 0.72 (p < 0.001), indicating high association. The ICC was 0.48 (p < 0.05), indicating fair agreement.

On the subscale level, we observed moderate to high correlations: r = − 0.58 (95% CI:  − 0.68;  − 0.47) for EQ-5D-5L mobility vs PROMIS physical function, r =  − 0.60 (95% CI:  − 0.70;  − 0.49) for EQ-5D-5L self-care vs PROMIS physical function, r = 0.65 (95% CI: 0.54; 0.74) for EQ-5D-5L pain/discomfort vs PROMIS pain interference, r = 0.70 (95% CI: 0.61; 0.78) for EQ-5D-5L anxiety/depression vs PROMIS depression, and r =  − 0.66 (95% CI:  − 0.74;  − 0.55) for EQ-5D-5L usual activities vs PROMIS ability to participate in social roles and activities.

**Fig. 1:**
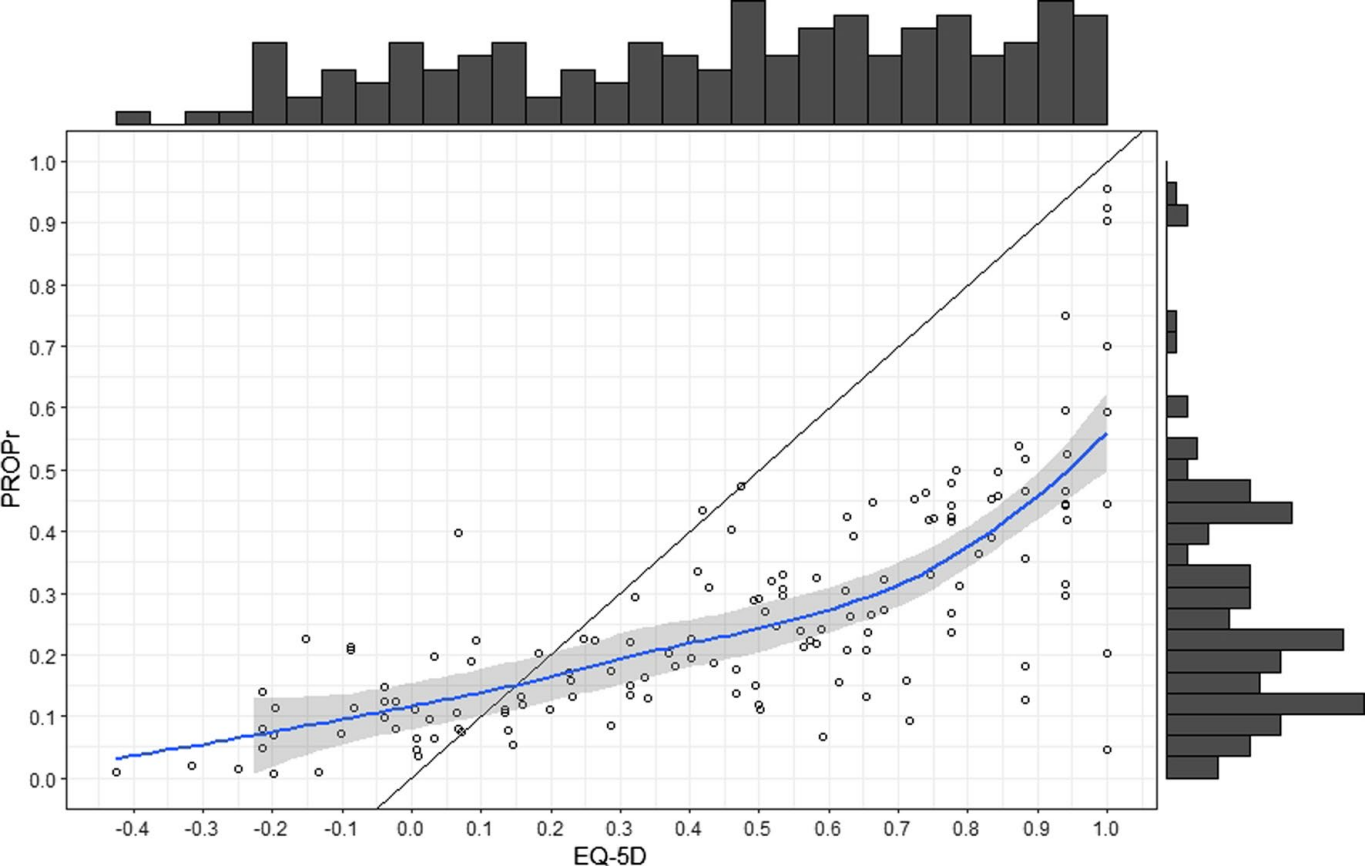
Scatterplot and histograms of PROPr and EQ-5D. The diagonal is the ideal line where correlation is 1

Scatterplots and histograms of the EQ-5D and the PROPr illustrate the positive correlation (Fig. 1). While the distribution of the PROPr was positively skewed, the distribution of the EQ-5D is negatively skewed: The mean PROPr was greater than the median PROPr (PROPr_Mean_ = 0.26 > PROPr_Median_ = 0.22) and the median EQ-5D was greater than the mean EQ-5D (EQ-5D_Median_ = 0.50 > EQ-5D_Mean_ = 0.44). The Pearson’s moment coefficient for skewness was γ = 1.25 > 0 for the PROPr and γ =  − 0.31 < 0 for the EQ-5D, also showing a positive skew for the PROPr and negative skew for the EQ-5D. The EQ-5D measurement range (− 0.425; 1.00) was wider than the PROPr measurement range (− 0.004; 0.955).

### Convergent validity

**Fig. 2:**
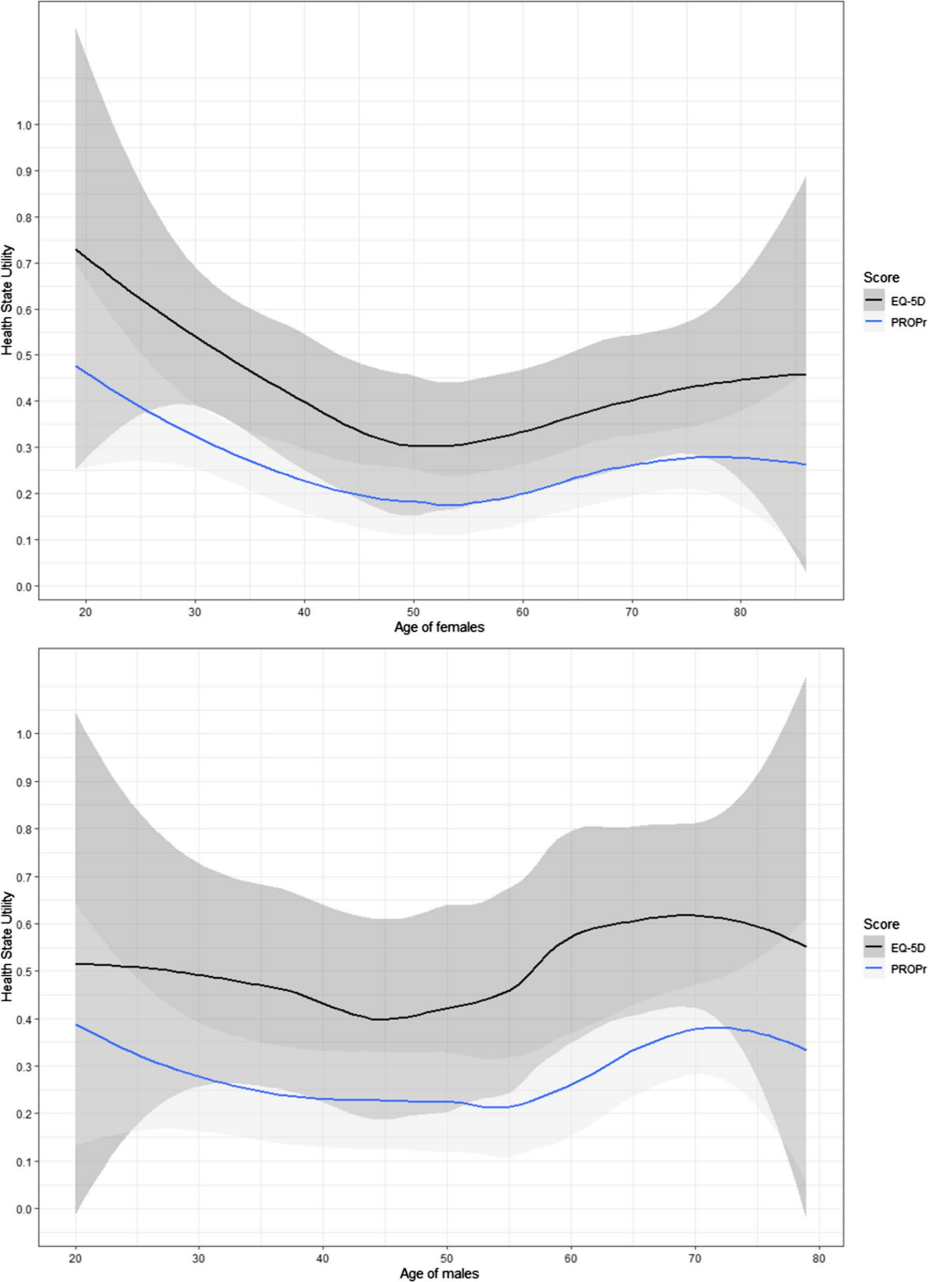
Health state utility measured in both EQ-5D and PROPr, dependent on sex and age. Lines are Loess smoothers. The light background represents confidence bands

Figure 2 illustrates the discrimination of the EQ-5D and the PROPr across sex and age. For both sex groups, the EQ-5D showed higher values than the PROPr. The PROPr’s narrower confidence intervals indicate that the scores are less scattered across the measurement range. Graphically, two scores showed similar but not fully parallel trends with large confidence intervals in both groups. As a statistical test, linear regression with interaction terms did not show significant interactions between instrument and age and sex (p < 0.05). Hence, the difference between the EQ-5D and PROPr seems to be caused by the instrument alone and not by age or sex.

**Fig. 3:**
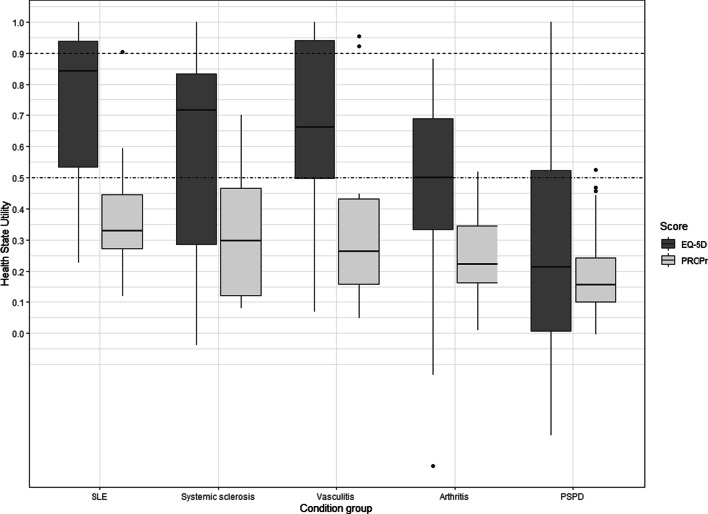
Health state utility measured in both EQ-5D and PROPr, dependent on condition. The dashed line at HSU = 0.9 refers to the estimated population average of the EQ-5D [33–37]; the dashed-dotted line at HSU = 0.5 refers to the estimated population average of the PROPr [18, 19]; SLE = systemic lupus erythematosus, PSPD = persistent somatoform pain disorder

Figure 3 illustrates how the two scores differentiate between the five most frequent conditions. The PROPr consistently measures HSU lower than the EQ-5D and has smaller confidence intervals. The hierarchy is the same: the best HSU is assigned to patients with SLE and the worst to patients with PSPD. Patients with systemic sclerosis had higher HSU than those with vasculitis and arthritis. A linear regression showed interaction terms for condition that were not statistically significant (p < 0.05) but nonetheless large, due to the small sample size.

The EQ-5D range is wider, with a difference of 0.62 from 0.84 in patients with SLE to 0.22 in patients with PSPD. The PROPr’s range between SLE (0.34) and PSPD (0.16) is only 0.18.

With the PROPr, for all conditions, the majority of the respective subsample scores a lower HSU than the population average of 0.5 [18, 19]. With the EQ-5D, a considerable share of patients with SLE and vasculitis do not show a lower HSU than the population average of 0.9. As no recent HRQoL data measured by the EQ-5D-5L (US value set) are available for the US general population, we used studies from comparable countries and the 3L value set as a comparator, assuming values would be similar [33–37].

### Ceiling and floor effects

Table 3 shows ceiling and floor effects on the subscale and score levels. At the subscale level, neither score shows significant floor effects, but the EQ-5D shows a significant ceiling effect. At the score level, the EQ-5D shows a significant ceiling effect (30.50%), while the PROPr shows a significant floor effect (41.84%).

**Table 3:** Proportions of cases observed below the 20th (floor effect) or above the 80th (ceiling effect) percentiles

		Floor effect (%)	Ceiling effect (%)
*Heath State Utility Score*			
PROPr		41.84	2.13
EQ-5D		2.13	30.50
*PROPr Subscales*	*EQ-5D Subscales*		
PROPr Cognition		1.42	19.15
PROPr Depression		4.26	15.60
	EQ-5D Anxiety/Depression	2.84	33.34
PROPr Fatigue		4.26	9.22
PROPr Pain Interference		3.55	8.51
	EQ-5D Pain/Discomfort	5.67	10.64
PROPr Physical Function		8.51	7.80
	EQ-5D Mobility	0.71	28.37
	EQ-5D Self-care	0.00	58.16
PROPr Sleep Disturbance		4.96	8.51
PROPr Ability to Participate in social roles and activities	9.22	8.51	
	EQ-5D Usual Activities	7.80	20.58
Average PROPr subscales		5.17	11.85
Average EQ-5D subscales		3.40	30.22

PROPr physical function shows a mild ceiling effect (8.51%), while the two corresponding EQ-5D dimensions, mobility and self-care, show high ceiling effects (28.37% and 58.16%, respectively). EQ-5D pain/discomfort and PROPr pain interference showed similar ceiling effects (10.64% and 8.51%). The comparison of ceiling effects of the depression subscales (33.34% vs 15.6%) and usual activities/ability to participate in social roles and activities (20.58% vs 14.18%) also favored PROPr subscales.

## Discussion

We investigated the measurement properties of the PROPr in a sample of patients with rheumatological and psychosomatic conditions and compared them to those of the EQ-5D.

Our first result confirms that the PROPr is a psychometrically valid instrument for assessing HSU in this patient group. Its psychometric properties of criterion and convergent validity are comparable with those in a general population sample [18, 19]. However, though correlation is high, the agreement – determined by the ICC – is only fair. The PROPr’s criterion validity is therefore limited, indicating that the PROPr conceptualizes HSU differently than the EQ-5D. The PROPr also had smaller confidence intervals, as it is less scattered across the measurement range. We attribute this to the fact that the PROPr uses two items per domain instead of just one, as the EQ-5D does.

Our second finding confirms that the PROPr yields a lower HSU than the EQ-5D. The mean difference was 0.18, which is smaller than that in the US general population (approximately 0.30). These differences were statistically invariant to age, sex, and condition. For age and sex, the interaction terms were small, though they were larger for conditions. Therefore, there could be an instrument effect in conditions that we were not able to detect. The original PROPr validation used the EQ-5D-3L crosswalk value set, while we used the 5L value set [18, 19]. Therefore, the mean difference between PROPr and EQ-5D is lower with the 5L value sets, while the correlation remains comparable. Many 5L value sets yield lower HSUs than the corresponding 3L crosswalk value sets [35]. Thus, the population average comparator might be lower than 0.9. The EQ-5D shows more considerable differences in only 3 of 5 conditions. The PROPr does so in all conditions, indicating higher sensitivity, as earlier results suggested [19].

Our third finding offers an explanation for the mean differences of the two scores: the PROPr shows no ceiling effect but a significant floor effect. Interestingly, while the PROPr moves from an approximate normal distribution with no floor effects in a general population sample to a positively skewed distribution with a floor effect in a patient sample, the EQ-5D is negatively skewed, showing ceiling effects in both the general population and patient samples [19, 25]. The floor effect of the PROPr (or PROMIS) domains was minor. This could potentially be resolved by choosing more sample-specific, i.e., easier items. But, because of the PROPr’s multiplicative MAUT function with its interactions and its lower boundary of 0, we would not expect that the PROPr’s floor effect could be resolved by a different set of items [16, 18]. Ceiling effects are a well-known issue for the EQ-5D that has improved by adding two levels from the 3L to the 5L version but still remains [34]. Using two items per domain, as the PROPr does, also contributes to preventing ceiling effects [38]. Our results suggest that the EQ-5D’s ceiling effect does not result from its valuation technique but from its dimension items. For example, the PROPr physical function domain outperforms the corresponding EQ-5D dimensions mobility and self-care in terms of ceiling effects. This means that, in order to achieve a high HSU of 0.9 or 1.0., the patient needs to be in better shape when measured by the PROPr than if measured by the EQ-5D, i.e. the PROPr’s upper anchor is higher. Note that our definition of ceiling/floor effects has the precondition that HSU is not uniformly distributed, which we could show is the case in our sample. Additionally, SG, the PROPr valuation method, yields 5–15% higher HSU than TTO, the EQ-5D valuation method, attributable to risk aversion [39]. Thus, the difference could be larger if valuation was performed with the same method and without limited ranges of measurement at either end. So, we can conclude that PROPr and EQ-5D seem to have different conceptualizations of HSU, probably due to their different construction, different number of domains and items, and their different valuation techniques.

Finally, we discuss the consequences on the ICER if the PROPr is used as the HSU score: first, the PROPr shows convergent validity, indicating that it is influenced by different subgroups the same way the EQ-5D is. However, the criterion measured is different from the EQ-5D as a HSU score for QALY, which is indicated by its fair agreement and therefore limited criterion validity. The PROPr’s low HSU scores are a result of an elevated upper anchor compared to the EQ-5D. This is problematic from a QALY perspective: In theoretical TTO terms, a mean PROPr of 0.5 in the general population as had been reported in earlier studies, means that a person is willing to trade half of his life duration to improve his current health status to optimal health. Or, put in SG terms, the average person would agree to a gamble to improve their current health state to perfect health with a chance of 50% of immediate death. Both statements seem unrealistic. Furthermore, as our results only apply to the PROPr measured with these 2 specific items per domain, it should be confirmed in future research if indeed the use of different and/or more items per domain, which is possible due to the IRT property, yield the same HSU scores. Summing up, the interpretation of EQ-5D QALY and PROPr QALY would therefore be very different and neither comparable nor interchangeable. Further attention should be devoted to the PROPr’s scaling and its impact on decision making when used for QALY in HTA.

Second, as a consequence, SOC health states would have a lower HSU with the PROPr. It could thus better distinguish improvements at the healthy end of the scale even in a smaller sample such as ours. For example, in our sample, the improvement of physical function could rather be measured with the PROPr than with the EQ-5D. Physical function is considered one of the key issues for rheumatological patients; therefore, an HSU score should capture its improvement [38, 40]. However, the PROPr may not capture the full extent of poor health, as the floor effect suggests. Consequently, the PROPr may not be able to capture small improvements for very sick patients who may be prioritized in HTA as they suffer more. Future research should investigate the PROPr’s validity in different levels of severity.

## Strengths and limitations

This study investigates the criterion and convergent validity and ceiling and floor effects of the PROPr in a patient sample, contributing to the knowledge about its measurement properties. A limitation is the small sample size, hardly reflecting the broad range of chronically ill patients. Therefore, our results need to be confirmed in other conditions and larger samples. Additionally, our data do not provide information about disease severity. Furthermore, this is a self-selected sample with a relatively high proportion of drop-outs and incomplete assessments, which might have led to selection bias. Future studies should aim to reduce the drop-out rate, such as by offering an incentive to respondents and lowering the response burden. Also, even though we used a sample of German patients, we had to use the US valuation of both scores to avoid systematic bias. A future valuation of the PROPr in a German sample is warranted to allow its use in German HTA.

## Conclusion

Our analysis confirms that the PROPr measures HSU considerably lower than the EQ-5D, which is a result of different construction, anchors and measurement ranges. The PROPr shows some favorable properties, such as the non-existence of ceiling effects and high correlational association but only fair agreement with the EQ-5D, and demonstrates a floor effect. Because the QALYs derived with EQ-5D are widely considered state-of-the-art, application of the PROPr for obtaining HSU scores for QALY measurements would be problematic. Future research is needed to discuss if the PROPr is eligible for QALY measurements.

## Supplementary Information

**Additional file 1:** Preliminary and final German version of PROPr items for cognition, fatigue, and ability to participate in social roles and activities. For original English version, see Table 1.

## Data Availability

The datasets used and/or analysed during the current study are available from the corresponding author on reasonable request.

## Abbreviations

3L: 3 Levels
5L: 5 Levels
CAT: Computerized adaptive testing
CI: Confidence interval
COPD: Chronic obstructive pulmonary disease
cTTO: Composite time trade-off
CUA: Cost-utility analysis
EQ-5D: EuroQoL 5 Dimensions 5 Level index value
HRQoL: Health-Related Quality of life
HSU: Health state utility
HTA: Health Technology Assessments
ICD-10: International Classification of Disease, 10th revision
ICER: Incremental Cost-Effectiveness Ratio
IRT: Item response theory
NICE: National Institute of Health and Clinical Excellence
M: Mean
MAUT: Multiattribute utility theory
Mn: Median
PHQ-9: Patient-Health Questionnaire-9
PROMIS: Patient Reported Outcome Measurement Information System
PROMIS-29 v2.0: PROMIS Profile 29 Version 2.0
PROPr: PROMIS Preference Score
PSPD: Persistent somatoform pain disorder
QALY: Quality-adjusted life years
SD: Standard deviation
SF-6D: Short Form-6 dimensions
SG: Standard gamble
SLE: Systemic lupus erythematosus
SOC: Standard of care
SS: Sjoegren’s syndrome
SSCl: Systemic sclerosis
TTO: Time Trade-off
u: Health state utility
US: United States of America
VAS: Visual Analogue Scale
