# Supplementary material for: Psychometric properties of the PROMIS Preference score (PROPr) in patients with rheumatological and psychosomatic conditions
Source: BMC Rheumatol. 2022 Mar 7;6:15. doi: 10.1186/s41927-022-00245-3 (PMC8898596; doi:10.1186/s41927-022-00245-3)
Supplement: Supplementary file 1 — Additional file 1: Preliminary and final German version of PROPr items for cognition, fatigue, and ability to participate in social roles and activities. For original English version, see Table 1. [file 41927_2022_245_MOESM1_ESM.docx]

**Appendix**

Table S1. Preliminary and final German versions of PROPr items for cognition, fatigue, and ability to participate in social roles and activities. For the original English version, see Table 1.

| PROPr domain | Item code | Preliminary version | Final version |
| --- | --- | --- | --- |
| Cognition | PC6r | Ich bin in der Lage gewesen, mich zu konzentrieren | Ich bin fähig gewesen, mich zu konzentrieren |
|  | PC27r | Ich bin in der Lage gewesen mich zu erinnern Dinge zu tun, wie meine Medizin einzunehmen oder etwas zu kaufen, was ich brauchte | Ich bin fähig gewesen, mich an Dinge zu erinnern, die ich tun musste, wie z.B. Medikamente einnehmen oder etwas kaufen, das ich benötigte |
| Fatigue | FATIMP21 | Wie oft waren Sie zu müde, ein Bad zu nehmen oder zu duschen? | Wie oft waren Sie zu müde, um zu baden oder zu duschen? |
| Ability to participate in social roles and activities | SRPPES31_CaPS | Es fällt mir schwer, mich um meine regelmäßigen persönlichen Verpflichtungen zu kümmern | Not yet finalized |
|  | SRPPER04_CaPS | Es fällt mir schwer, an Freizeitaktivitäten mit anderen teilzunehmen | Not yet finalized |
